# Deletion of α-neurexin II results in autism-related behaviors in mice

**DOI:** 10.1038/tp.2014.123

**Published:** 2014-11-25

**Authors:** J Dachtler, J Glasper, R N Cohen, J L Ivorra, D J Swiffen, A J Jackson, M K Harte, R J Rodgers, S J Clapcote

**Affiliations:** 1 School of Biomedical Sciences, University of Leeds, Leeds, UK; 2 School of Pharmacy and Pharmaceutical Sciences, University of Manchester, Manchester, UK; 3 Institute of Psychological Sciences, University of Leeds, Leeds, UK

## Abstract

Autism is a common and frequently disabling neurodevelopmental disorder with a strong genetic basis. Human genetic studies have discovered mutations disrupting exons of the *NRXN2* gene, which encodes the synaptic adhesion protein α-neurexin II (Nrxn2α), in two unrelated individuals with autism, but a causal link between *NRXN2* and the disorder remains unclear. To begin to test the hypothesis that Nrxn2α deficiency contributes to the symptoms of autism, we employed Nrxn2α knockout (KO) mice that genetically model Nrxn2α deficiency *in vivo*. We report that Nrxn2α KO mice displayed deficits in sociability and social memory when exposed to novel conspecifics. In tests of exploratory activity, Nrxn2α KO mice displayed an anxiety-like phenotype in comparison with wild-type littermates, with thigmotaxis in an open field, less time spent in the open arms of an elevated plus maze, more time spent in the enclosure of an emergence test and less time spent exploring novel objects. However, Nrxn2α KO mice did not exhibit any obvious changes in prepulse inhibition or in passive avoidance learning. Real-time PCR analysis of the frontal cortex and hippocampus revealed significant decreases in the mRNA levels of genes encoding proteins involved in both excitatory and inhibitory transmission. Quantification of protein expression revealed that Munc18-1, encoded by *Stxbp1*, was significantly decreased in the hippocampus of Nrxn2α KO mice, which is suggestive of deficiencies in presynaptic vesicular release. Our findings demonstrate a causal role for the loss of Nrxn2α in the genesis of autism-related behaviors in mice.

## Introduction

Autism is a widespread cognitive disorder characterized by impairments in social interactions, communication and language development, that can be accompanied by stereotyped patterns of behavior. Autism is a highly heritable disorder with concordance rates as high as 90% for monozygotic twins,^1^ but the underlying molecular and neuropathophysiological basis is unknown in most cases. However, recent genetic and genomic studies have implicated a large number of genes in autism,^2^ many of which encode synaptic proteins,^3^ indicating that synaptic dysfunction may have a critical role in autism.

The neurexins are a family of synaptic adhesion proteins encoded by paralogous genes (*NRXN1*-*3*) that have a key role in synaptic function. Each gene is transcribed in neurons from two independent promoters to yield longer (α) proteins with six laminin/neurexin/sex hormone (LNS) binding domains, and shorter (β) proteins with one LNS binding domain. Intracellularly, α-neurexin binds to CASK, Mint, Munc18, syntenin and synaptotagmin, suggesting a role in vesicular release.^4, 5, 6^ Postsynaptic binding with PSD-95 or gephyrin via neuroligins (also associated with autism^7^), leucine-rich repeat transmembrane proteins (LRRTMs) or dystroglycan can directly influence NMDA, AMPA or GABAergic receptors at the synapse, thereby altering a cell's excitatory or inhibitory tone.^8, 9, 10^ The promoter for α-neurexin II (Nrxn2α) transcripts lies upstream of *NRXN2* exon 1, whereas the promoter for β-neurexin II is located in the intron downstream of exon 17.^11^ The first evidence for a potential role of *NRXN2* in autism was provided by a report of a frameshift mutation within *NRXN2* exon 12 in a boy with autism and his father with severe language delay.^12^ This mutation results in a truncated Nrxn2α protein that lacks the binding sites for the established postsynaptic binding partners LRRTM2 and neuroligin-2, but does not affect β-neurexin II.^12^ Subsequently, a 21-year-old man with a clinical phenotype including autistic traits, such as speech and language deficits and insistence on routine, was reported to have a 570-kb *de novo* deletion of 24 genes at chromosome 11q13.1, including *NRXN2*.^13^ However, a clear causal relationship between *NRXN2* and autism has not been established.

To begin to test the hypothesis that Nrxn2α deficiency contributes to the symptoms of autism, we employed mice with a targeted mutation (*Nrxn2*^*tm1Sud*^; MGI:3042719) that deletes the first exon of *Nrxn2* and abolishes expression of Nrxn2α, but does not affect β-neurexin II.^14^ The 30-day survival rate and gross brain anatomy of Nrxn2α null mutants are unaltered compared with wild-type littermates.^14, 15^ In light of the putative link between Nrxn2α and autism—diagnosis of which is based purely on behavioral assessment—we predicted that Nrxn2α knockout (Nrxn2α KO) mice might exhibit autism-relevant behavioral abnormalities. Herein, we report that Nrxn2α KO mice displayed altered anxiety-like and social behaviors consistent with a causal role for the loss of Nrxn2α in the genesis of autism-related behaviors.

## Materials and methods

All the procedures were approved by the University of Leeds Animal Ethical and Welfare Review Board and were performed under the UK Home Office Project and Personal Licences.

### Animals

B6;129-*Nrxn3*^*tm1Sud*^*/Nrxn1*^*tm1Sud*^*/Nrxn2*^*tm1Sud*^/J mice (JAX #006377) were purchased from the Jackson Laboratory (Bar Harbor, ME, USA) as heterozygous KO at *Nrxn1*, homozygous KO at *Nrxn2* and wild-type at *Nrxn3*. We subsequently outbred to the C57BL/6NCrl strain (Charles River, Margate, UK) to obtain mice that were Nrxn2α KO heterozygotes, but wild-type at *Nrxn1* and *Nrxn3*. Nrxn2α KO heterozygotes were then intercrossed to obtain wild-type (WT) and KO littermates. DNA extracted from ear biopsies was used for PCR-based genotyping according to the Jackson Laboratory *Nrxn1* v5, *Nrxn2* v5 and *Nrxn3* v1 protocols (http://jaxmice.jax.org/strain/006377.html#genotype). Briefly, the primers 5′-GAGATGGAGAGCCAGACACC-3′ (common forward), 5′-CAGTGCCATGGACTCAGTAGC-3′ (WT reverse) and 5′-GCATCGCATTGTCTGAGTAGG-3′ (KO reverse) were used with HotShot Diamond (Clent Life Science, Stourbridge, UK) using the thermocycling program of: 94 °C for 5 min, followed by 35 cycles of 94 °C for 30 s, 64 °C for 60 s and 72 °C for 60 s, followed by 72 °C for 120 s. PCR products were visualized using electrophoresis, with a 190-bp band indicating the WT allele, and a 310-bp band indicating the KO allele.

Litters were separated by sex at postnatal day 21, when mice were housed with at least one other mouse of the same sex and age, with a maximum of five mice per cage. Food and water were provided *ad libitum*, except for the buried food experiment. Lighting was provided in a 12:12 dark/light cycle, with the light cycle commencing at 06:00.

### Behavioral testing

All behavioral experiments were conducted using young adults over 8 weeks of age. All the mice were extensively handled before testing. WT and KO mice were tested in the following behavioral experiments (in order): open field, elevated plus maze (EPM), forced-swim test, social interaction, emergence test, novel object exploration, prepulse inhibition (PPI), passive avoidance. For detailed methodology, see Supplementary Materials and Methods.

### Quantitative RT-PCR

WT (*n*=5) and Nrxn2α KO (*n*=5) mice were killed by CO_2_ asphyxiation and their brains were quickly extracted and snap frozen in liquid N_2_. Frontal cortex and hippocampus were dissected on ice, and tissue was stored in RNAlater (Ambion, Paisley, UK) at 4 °C for up to 7 days. RNAlater was removed and tissue was homogenized in 1 ml TRIzol Reagent (Invitrogen, Paisley, UK). RNA was extracted from the homogenate using a PureLink RNA Kit (Ambion), followed by spectrophotometric analysis of purity/integrity by 260 nm analysis of RNA concentration and 260/280 nm ratio analysis of RNA purity. Two microlitres of each RNA sample were converted into cDNA using a Quantitect reverse transcription kit (Qiagen, Manchester, UK). The cDNA was stored at −20 °C before analysis by quantitative RT-PCR. A total of 0.1 μg of cDNA (in triplicate) was used to quantify gene expression with a Quantitect SYBR Green quantitative RT-PCR kit (Qiagen) using the thermocycling program of: 95 °C for 15 mins, followed by 40 cycles of 94 °C for 15s, 55 °C for 30 s and 72 °C for 30s.

All the data were normalized to a *Cyc1* neuronal reference gene. To select an optimal reference gene, the stability of four genes commonly used in real-time RT-PCR studies (*Actb* (β-actin), *Cyc1*, *Pgk1* and *Rpl13a*) was tested. Three different samples (with two replicates per sample) per genotype and brain area were run for each gene. Normfinder software^16^ was used with the obtained cycle threshold (Ct) values to calculate the expression stability of the four genes. *Cyc1* was consistently the most stable gene in both frontal cortex and hippocampus. Using a combination of genes did not substantially improve the stability (data not shown).

Thirteen transcripts in total were studied: parvalbumin (*Pvalb*), GAD_65_ (*Gad2*), GAD_67_ (*Gad1*), AMPA receptor subunit 1 (*Gria1*), NMDA receptor subunit 1 (*Grin1*), NMDA receptor subunit 2a (*Grin2a*), NMDA receptor subunit 2b (*Grin2b*), postsynaptic density protein 93 (*Dlg2*), postsynaptic density protein 95 (*Dlg4*), syntaxin-binding protein 1 (*Stxbp1*), Homer protein 1 (*Homer1*), vesicular glutamate transporter (*Slc17a7*), and vesicular inhibitory amino-acid transporter (*Slc32a1*). Primers for *Stxbp1*, *Grin2b*, *Homer1*, *Pgk1*, *Rpl13a* and *Cyc1* were designed using the Roche Universal Probe Library ProbeFinder version 2.50 (Table 1) and were synthesized by Sigma (Haverhill, UK). The remaining primers were QuantiTect Primer Assays purchased from Qiagen.

Analysis was carried out using the 2^-ΔΔCt^ method^17^ and data are displayed as relative quantification values, relative to WT levels.

### Western blotting

WT (*n*=4) and *Nrxn2* KO mice (*n*=4), different to those used for the RT-PCR, were killed by CO_2_ asphyxiation and their brains were quickly extracted, divided into hemispheres, snap frozen in liquid N_2_ and subsequently stored at −80 °C. The cortex and hippocampus were dissected under a microscope and homogenized at a concentration of 333 mg ml^−1^ in RIPA Lysis Buffer with 0.5% sodium orthovanadate, 0.5% PMSF, 0.5% protease inhibitor cocktail (Santa Cruz, Heidelberg, Germany) and 1 × Phos-STOP (Roche, Welwyn Garden City, UK) on ice. The homogenate was centrifuged at 4 °C, the supernatant was aliquoted and protein concentration was measured by Bradford assay. Samples were stored at −80 °C. Aliquots of 30 μg total protein were prepared for loading by the addition of Laemmli sample buffer (Bio-Rad, Hemel Hempstead, UK) with 5% β-mercaptoethanol and incubated at 95 °C for 5 min.

Samples were subjected to gradient SDS–polyacrylamide gel electrophoresis (100 V, 1.5 h) on polyacrylamide gels (4–15%) (Mini-PROTEAN TGX, Bio-Rad), transferred to BioTrace PVDF transfer membranes (Pall, Portsmouth, UK) (100 V 1.5 h on ice), and blocked for either 1–2 h at room temperature or overnight at 4 °C in 5% skimmed milk in 1 × phosphate-buffered saline with 0.05% Tween-20. Membranes were incubated with primary antibodies in 5% milk for 1 h at room temperature or 4 °C overnight at the following concentrations: Munc18-1 (sc-14557; Santa Cruz) 1:1000; parvalbumin (SAB4200545; Sigma) 1:1000; PSD-95 (sc-32290; Santa Cruz) 1:1000; GluN2A (sc-31542; Santa Cruz) 1:100. Anti-goat (sc-2020; Santa Cruz) and anti-mouse (sc-2371; Santa Cruz) HRP-linked secondary antibodies were incubated in 5% milk for 1 h at room temperature. Bound peroxidase-conjugates were visualized using ECL western blotting substrate (Promega, Southampton, UK). To confirm equal loading, membranes were immersed in stripping buffer (69 mM SDS, 63 mM Tris, 0.7% β-mercaptoethanol, pH 6.8) at 50 °C for 30 min before incubating with anti-β-actin (A1978; Sigma, Poole, UK) 1:5000. All western blots were repeated a minimum of three times. Densitometry was performed using ImageJ (v1.46; http://imagej.nih.gov/ij), with expression normalized to the β-actin loading control.

### Data analysis

All the data are expressed as mean±s.e.m. To assess the differences between the variables and their impact upon performance, two-sample *t*-tests or analyses of variance were conducted. Performance across time bins was analyzed by repeated measures analysis of variance. If there were significant interactions between variables, tests of simple main effects were performed (Bonferroni corrected), followed by *post hoc* analysis where necessary. All analyses were performed using SPSS version 20. In all cases, α was set at ⩽0.05. Graphs were drawn using GraphPad Prism version 6. Statistical significance within the figures is represented as: ***P<0.0001, **P<0.01 and *P<0.05.

## Results

### Nrxn2α KO mice display deficits in social behavior

**Figure 1:**
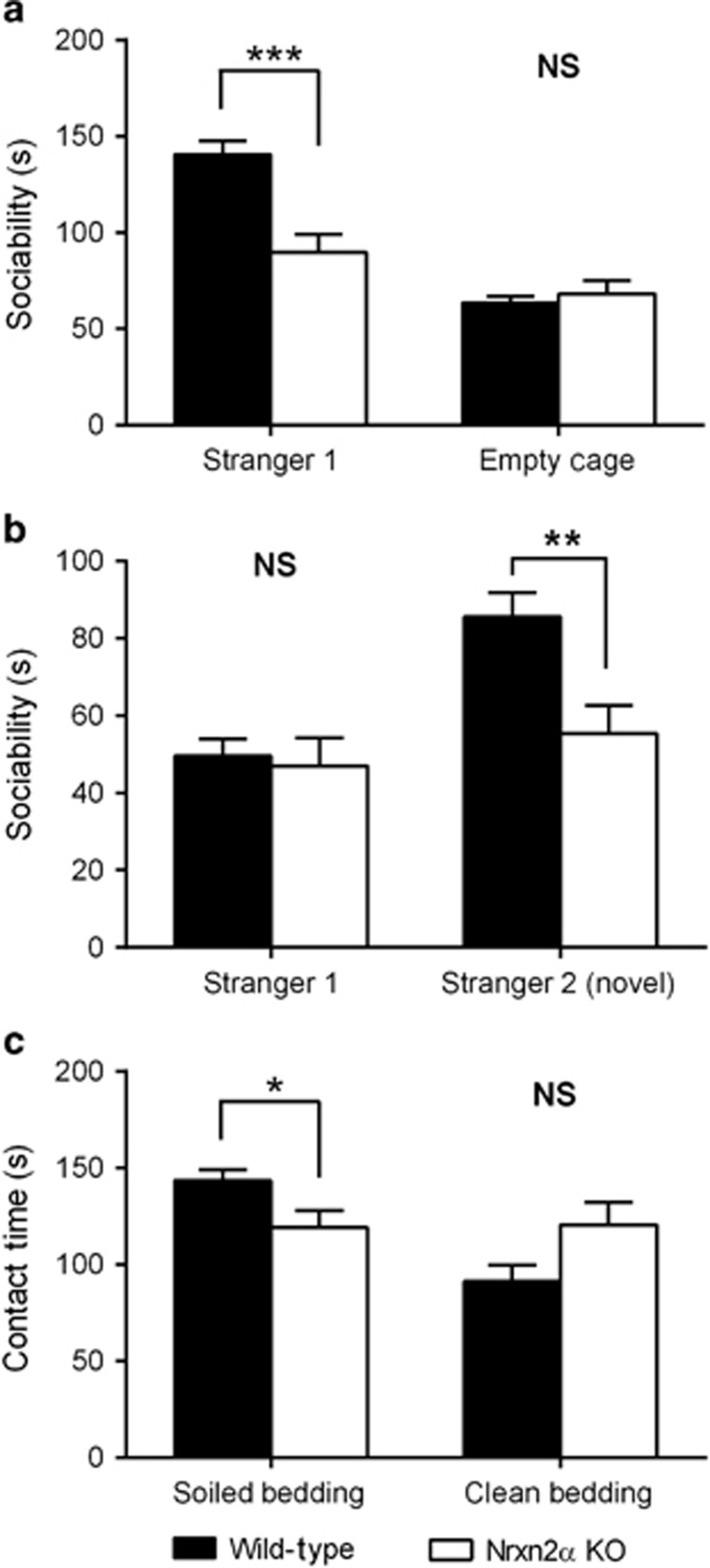
Nrxn2α KO mice exhibit deficits in sociability and social memory. During the first phase (**a**), whereby the test mouse had to discriminate between a novel mouse and an empty but identical cage, Nrxn2α KO mice (*n*=16) display no significant preference for exploring the novel mouse compared with the empty cage, whereas WT (*n*=31) showed a very clear discrimination (repeated measure analysis of variance (RM ANOVA), significant genotype × discrimination interaction; F_(1,45)_=14.89, *P*<0.0001). Tests of simple main effects found a significant effect of genotype when exploring the novel mouse (F_(1,45)_=18.51, *P*<0.0001) but not the empty cage (F_(1,45)_<1, *P*>0.05). In stage 2 (**b**), the preference of the test mouse to discriminate between the previously explored mouse (Stranger 1) and a second novel mouse (Stranger 2) was measured. Nrxn2α KO mice spent a similar time as WT exploring Stranger 1, but showed significantly less exploration of Stranger 2 (RM ANOVA, significant genotype × discrimination interaction; F_(1,45)_=8.08, *P*=0.007). Tests of simple main effects confirmed a significant effect of genotype on time exploring the novel mouse (F_(1,45)_=8.92, *P*=0.005) but not on time exploring the previously explored mouse (F_(1,45)_<1, *P*>0.05). Nrxn2α KO mice were also unable to discriminate between exploring soiled vs clean bedding (**c**). Nrxn2α KO mice (*n*=13) showed no preference for either cage, whereas WT (*n*=11) spent a proportionately longer time exploring the cage containing the soiled bedding (RM ANOVA, significant genotype × discrimination interaction; F_(1,22)_=8.37, *P*=0.008). Tests of simple main effects found a significant effect of genotype on time exploring the soiled bedding (F_(1,22)_=5.01, *P*=0.036) but not the clean bedding (F_(1,22)_=3.81, *P*>0.05). KO, knockout; Nrxn2α, α-neurexin II; NS, not significant; WT, wild type. *P<0.05; **P<0.01; ***P<0.0001.

In view of the putative link between Nrxn2α and autism, we assessed whether Nrxn2α KO mice with a predominantly C57BL/6 genetic background exhibit autism-related behavioral abnormalities. As impaired sociability is one of the core diagnostic criteria for autism,^18^ we examined the social interaction of Nrxn2α KO mice in a three-chambered assay for sociability, in which mice were given a choice between spending time in the side with an unfamiliar mouse enclosed in a wire cage (Stranger 1) or in the side with an empty wire cage.^19^ Unlike their WT littermates, Nrxn2α KO mice failed to show a significant preference for the unfamiliar conspecific. However, both genotypes spent an equivalent amount of time in proximity to the empty cage (Figure 1a). Following the sociability assay, subjects were given a test for social novelty preference, with a choice between the original unfamiliar mouse (Stranger 1) and a new unfamiliar mouse (Stranger 2). WT mice showed a clear preference for exploration of Stranger 2, whereas no such preference was shown by Nrxn2α KO mice, although both genotypes spent a similar time in proximity to Stranger 1 (Figure 1b). There was no genotypic difference in general ambulation in the three-chambered arena, as Nrxn2α KO and WT mice traveled similar distances during each phase of testing (Supplementary Figure S1). To determine whether the reduced social exploration time in Nrxn2α KO mice was related to potential anxiety caused by the presence of a novel conspecific, we tested the preference for exploring soiled versus clean bedding in the same three-chambered arena using a previously untested cohort of mice. WT mice spent a greater proportion of time in proximity to the cage that contained the soiled bedding, compared with that containing the clean bedding, whereas Nrxn2α KO mice showed no bias towards either cage (Figure 1c). To test whether the lack of sociability could be related to a potential deficit in olfaction in the Nrxn2α KO mice, we examined their ability to locate buried food (Supplementary Figure S1d). There was no significant difference between the genotypes in latency to find the food. Three KO mice required the full length of the experiment to find the food, but two of these had previously shown a preference for exploring soiled bedding rather than clean bedding, suggesting that an olfactory deficit is unlikely.

### Nrxn2α KO mice display increased anxiety in tests of exploratory activity

In addition to the core symptoms of the disorder, autism is characterized by a high prevalence of all diagnostic subtypes of anxiety.^20^ Indeed, anxiety-related dysfunction can often be as significant as, or even greater than, the difficulties arising from the core symptoms.^21^ Anxiety in Nrxn2α KO mice was assessed in four tests of exploratory activity: the open field, EPM, emergence and novel object tests.

**Figure 2:**
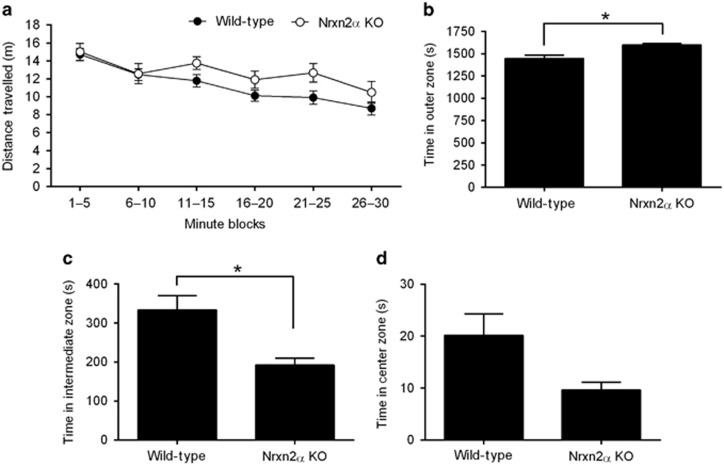
Activity of Nrxn2α KO mice in the open field. (**a**) Nrxn2α KO mice (*n*=16) display a marginal but non-significant increase in locomotion compared with WT mice (*n*=33) over 30 min of free exploration (two-way repeated measures analysis of variance, main effect of time block (F_(5,235)_=18.12, *P*<0.0001), no effect of genotype (F_(1,47)_=2.44, *P*>0.05) or interactions (F_(5,235)_<1, *P*>0.05)). During the trial, the arena floor was divided into three zones (outer, intermediate, center) and the mice were tracked automatically. Nrxn2α KO mice spent significantly more time within the outer zone (**b**; *t*_(47)_=2.54, *P*=0.015; thigmotaxis) and significantly less time in the intermediate zone (**c**; *t*_(47)_=2.59, *P*=0.013). There was also a trend for Nrxn2α KO mice to spend less time in the center zone (**d**; *t*_(47)_=1.76, *P*=0.085). KO, knockout; Nrxn2α, α-neurexin II; WT, wild type. *P<0.05.

The open field is a measure of anxiety dependent upon the natural aversion of mice for a novel, brightly lit open arena. In this situation, mice spontaneously prefer the periphery to activity in the central parts of the open field. This wall-hugging tendency, known as thigmotaxis, is bidirectionally sensitive to anxiogenic (increased) and anxiolytic (decreased) drugs, and is used as a measure of anxiety in mice.^22^ Over 30 min of free exploration in the open field, the total distance traveled was not significantly different between Nrxn2α KO and WT mice (Figure 2a). However, Nrxn2α KO mice spent significantly more time in the peripheral zone near the walls (Figure 2b), significantly less time in an intermediate zone (Figure 2c) and showed a trend approaching significance (*P*=0.085) for less time in the center of the arena (Figure 2d), but there were no genotypic differences in the total number of zone entries (Supplementary Figures S2a and c). Nrxn2α KO mice also spent more time rearing than WT mice, but there was no genotypic difference in the amount of time spent self-grooming (Supplementary Figures S2d and e).

**Figure 3:**
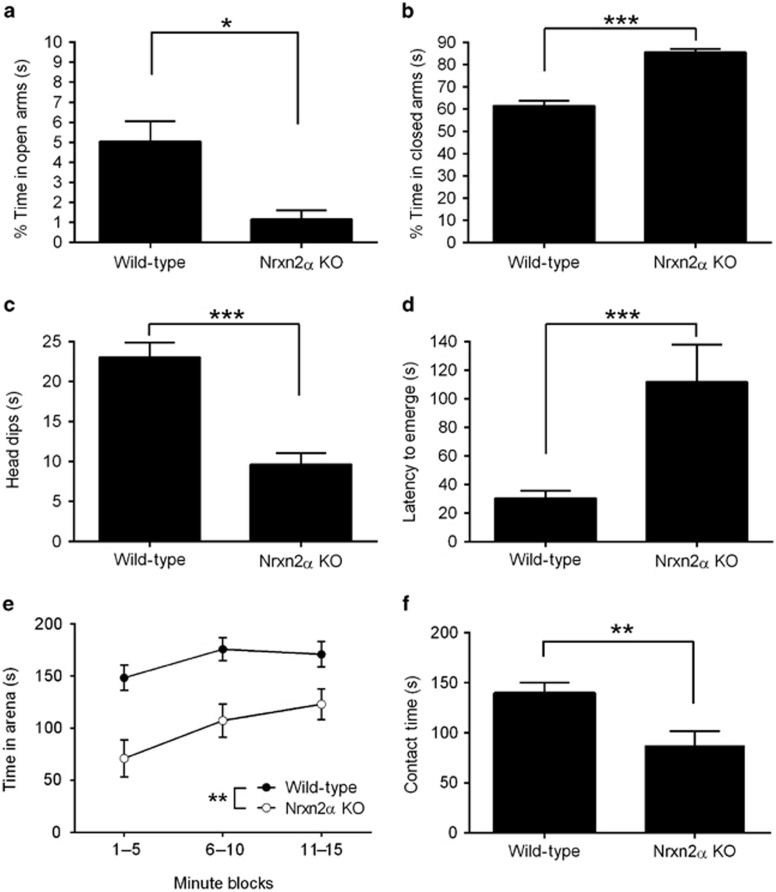
Nrxn2α KO mice show an anxiety-like phenotype. In the elevated plus maze, KO mice (*n*=16) spent significantly less time in the open arms (**a**; *t*_(47)_=2.62, *P*=0.012) and significantly more time in the closed arms (**b**; *t*_(47)_=6.84, *P*<0.0001) compared with WT (*n*=33). KO mice also make significantly fewer head dips (**c**; *t*_(47)_=4.68, *P*<0.0001). In the emergence test, the latency to emerge from an enclosed shelter into an open arena was significantly longer in Nrxn2α KO mice (*n*=16) compared with WT (*n*=33) (**d**; *t*_(47)_=4.16, *P*<0.0001) and, overall, they spent significantly less time out of the enclosed shelter over the 15 min trial (**e**; two-way repeated measures analysis of variance, main effect of time block (F_(2,94)_=14.34, *P*<0.0001) and genotype (F_(1,47)_=12.30, *P*=0.001), no significant interactions (F_(2,94)_=2.01, *P*>0.05)). In a familiar environment, Nrxn2α KO mice also spent significantly less time engaging with novel objects (**f**; *t*_(47)_=2.86, *P*=0.006). KO, knockout; Nrxn2α, α-neurexin II; WT, wild type. *P<0.05; **P<0.01; ***P<0.0001.

The EPM test exploits the conflict between the tendency of mice to investigate a novel environment, and to avoid brightly lit open areas. In this test, Nrxn2α KO mice spent significantly less time in the open arms (Figure 3a) and more time in the closed arms (Figure 3b) than WT littermates. Nrxn2α KO mice also made significantly fewer exploratory head dips from the center and open arms (Figure 3c) and spent significantly less time on the central square (Supplementary Figure S3a). Although Nrxn2α KO mice made significantly fewer total entries, and traveled significantly less overall than WT mice (Supplementary Figures S3b and e), it is unlikely that hypoactivity alone can explain their EPM behavior, as their ambulation in the other tests of exploratory activity was unaltered.

In the emergence test, mice were placed inside a small enclosure, and evaluated for the time taken to emerge from it into a larger, brightly lit open arena. Emergence latencies reflect anxiety levels, being shorter in rodents injected with diazepam.^23^ Nrxn2α KO mice took a substantially (3.7 times) longer time than WT mice to emerge from the enclosure (Figure 3d). Over the 15 min trial, Nrxn2α KO mice also spent significantly more time in the enclosure (Figure 3e), and made significantly fewer entries into the open arena (Supplementary Figure S3f) than WT mice.

State anxiety is a transient emotional response related to exposure to a threatening stimulus, whereas trait anxiety is an enduring feature determining propensity for anxiety.^24^ The EPM and open field tests have been described as measures of state anxiety, whereas the novel object test in a familiar environment is proposed to assess trait anxiety.^24, 25^ In the novel object test, Nrxn2α KO mice spent significantly less time than WT littermates exploring a novel object (Figure 3f). However, locomotor activity was similar between genotypes during both the habituation and test phases (Supplementary Figures S3g and h).

To assess depression-related behaviors in Nrxn2α KO mice, we used the Porsolt forced-swim test and the tail suspension test, both of which involve measurement of escape attempts and behavioral despair. In each of these tests, Nrxn2α KO and WT mice were statistically indistinguishable (Supplementary Figures S4a and b).

### Nrxn2α KO mice exhibit normal PPI and passive avoidance learning

PPI is a robust operational measure of sensorimotor gating, a process important for filtering extraneous sensory information from the external environment. In the few studies conducted to date, autism patients have not shown consistent deficits in PPI,^26, 27^ with only one study reporting decreased PPI in autistic subjects under specific testing conditions.^28^ We found that Nrxn2α KO and WT mice showed similar startle responses to varying intensities of sound, and similar magnitudes of PPI (Supplementary Figures S4c and d), thereby suggesting normal sensorimotor gating in Nrxn2α KO mice.

Up to 40% of individuals with autism have IQ scores low enough (<35) to be classified within the range of severe-to-profound intellectual disability.^29^ Conversely, a man with speech problems, autistic traits and deletion of the whole *NRXN2* gene was reported to have an IQ of 113 in a nonverbal intelligence test, suggesting that his mental impairment was primarily restricted to speech and language.^13^ Therefore, we examined long-term (24-h) memory in Nrxn2α KO mice using step-through passive avoidance, a fear-motivated test that requires the subject to refrain from entering a specific environment (a dark chamber) in which an aversive stimulus (a mild electric shock) has previously been experienced. We found that Nrxn2α KO and WT mice had similar retention latencies 24 h after the electric shock was given (Supplementary Figure S4e), indicating normal cognitive performance in this hippocampus-dependent test.^30^

### Nrxn2α KO mice show a decrease in hippocampal Munc18-1

**Figure 4:**
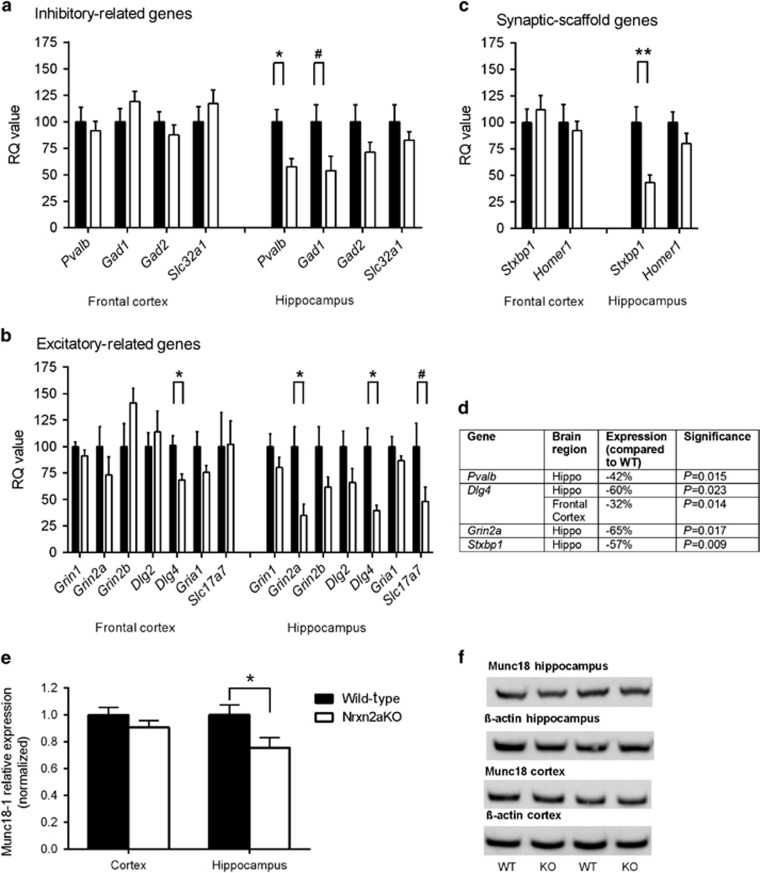
Altered mRNA transcript and protein expression levels in the frontal cortex and hippocampus of Nrxn2α KO brain. The 13 genes examined were divided into groups of inhibitory-related (**a**), excitatory-related (**b**) and synaptic scaffold-related (**c**). Within the frontal cortex, Nrxn2α KO mice showed significant reductions in the mRNA level of *Dlg4*, whereas in the hippocampus, *Pvalb*, *Grin2a*, *Stxbp1* and *Dlg4* were also reduced. *Gad1* and *Slc17a7* (VGlut1) both had reductions approaching significance (note in **a** and **b**, ^#^*P*=0.059 and ^#^*P*=0.086, respectively). (**d**) Summary of the significantly altered genes (unpaired *t*-test). (**e** and **f**) Within the hippocampus, western blotting confirmed a decrease in the protein expression of Munc18-1 (*Stxbp1*) (*t*_(22)_=2.31, *P*=0.031), although there was no significant difference in the cortex (*P*>0.05). KO, knockout; Nrxn2α, α-neurexin II; RQ, relative quantification; WT, wild type. *P<0.05.

Mice with deletion of other genes implicated in autism have shown differences in the level of synaptic proteins,^31^ so we used real-time RT-PCR analysis to measure the mRNA levels of 13 genes encoding synaptic proteins to ascertain whether their expression was altered by Nrxn2α deficiency. These genes were chosen on the basis of either known direct interactions with neurexin at the presynapse (for example, *Stxbp1*) or indirectly via neuroligins at the postsynapse (for example, *Dlg4*, *Pvalb*). We examined mRNA levels in two brain regions: the frontal cortex and hippocampus, both of which have links to autism.^32, 33^ *Dlg4,* encoding PSD-95, was the only transcript tested that had altered mRNA levels in both the frontal cortex and hippocampus, with expression significantly decreased in Nrxn2α KO mice. In the hippocampus, the mRNA levels of genes that encode proteins involved in both inhibitory (*Pvalb*; parvalbumin; Figure 4a) and excitatory (*Grin2a*; NMDA receptor subunit 2a; Figure 4b) transmission were significantly decreased in Nrxn2α KO mice. The mRNA level of *Stxbp1*, encoding Munc18-1, was also significantly reduced in the Nrxn2α KO hippocampus (Figure 4c). Munc18-1 has been shown to interact presynaptically with neurexins to facilitate presynaptic vesicular release.^34^

To determine whether these transcriptional changes led to detectable changes in protein abundance, we tested homogenates of frontal cortex and hippocampus by western blotting. Within the hippocampus, there was a significant reduction in the abundance of Munc18-1 (Figures 4e and f), but there was no significant difference in the frontal cortex. None of the other genes with significantly different mRNA levels in Nrxn2α KO mice showed detectable differences in protein abundance (Supplementary Figure S5).

## Discussion

Although there has been increasing focus upon the etiology of autism, its genetic basis remains poorly defined. Despite this, an increasing body of evidence has implicated the neurexin gene family in autism. Although deletions of *NRXN1* are associated with autism,^35^ recent studies have also discovered deletions affecting *NRXN2* in autism patients,^12, 13^ although a causative link between *NRXN2* and autism has not been established. In the present study, we found that deletion of the Nrxn2α gene in mice can replicate some of the core symptoms of autism. We found that Nrxn2α KO mice show reduced sociability, while also exhibiting an anxiety phenotype in the open field, EPM and emergence tests. Following quantification of mRNA extracts and protein expression, we found that deletion of Nrxn2α is associated with decreased expression of the presynaptic protein Munc18-1, which may potentially contribute to the altered behavioral state of Nrxn2α KO mice.

The diagnosis of autism in humans is made upon assessment of aberrant behavioral phenotypes, typically social, communication, repetitive and stereotyped behaviors. We found that Nrxn2α KO mice fail to show sociability with novel conspecifics or a preference for exploring social odors. This behavioral phenotype is thus consistent with one of the core symptoms of autism. A similar phenotype is shown by *Shank3* KO mice, which also genetically model a mutation found in autism, with deficits in sociability and social recognition.^35^ However, given the lack of initial sociability in Nrxn2α KO mice, it is difficult to determine to what extent social memory was actually affected. In contrast, mice null for another gene in the neurexin family, α-neurexin I (Nrxn1α), have shown heightened sociability, with significantly more time spent exploring the stranger mouse in the three-chamber social approach test and more aggression towards juvenile conspecifics,^36^ although another study observed unaltered sociability.^37^ Nrxn2α KO mice may thus model social deficits associated with autism better than Nrxn1α KO mice. Although it is conceivable that the generalized anxiety phenotype of Nrxn2α KO mice could have influenced their performance in the three-chamber social approach test, the similar total locomotion of Nrxn2α KO and WT mice across all phases of the test indicates that there was not an effect of hypoactivity (Supplementary Figure S1).

Nrxn2α KO mice did not model other core symptoms of autism in the behavioral tests that we carried out. Within the open field, they did not exhibit stereotyped repetitive behaviors, as have been observed in *Shank3* KO mice,^38, 39^ 16p11.2 deletion mice^40^ and Nrxn1α KO mice.^37^ Nrxn2α KO mice may simply not exhibit this phenotype, or the anxiogenic effect of the open field may have reduced the chance of observing repetitive behaviors. Altered communication is also a hallmark of autism. Changes in ultrasonic vocalization have been found in *Shank3* KO mice,^39^ but we did not test for this phenotype.

Autism is frequently comorbid with reduced intellectual ability.^41^ To assess long-term memory, we used the fear-motivated passive avoidance test, but found no impairment in Nrxn2α KO mice. It is possible that Nrxn2α deletions do not directly impair intellectual capacity, as the male patient with a whole gene deletion of *NRXN2* had an IQ of 113, although he did have deficiencies in speech and language.^13^ Similarly, Nrxn1α KO mice did not show cognitive impairments in the Morris water maze.^37^ However, as Nrxn1α deletions have been found in mentally retarded subjects without autism,^42^ further work is warranted to understand the role of the neurexins in cognitive processes.

We observed an anxiety phenotype in Nrxn2α KO mice across several different paradigms. In autism patients, it has been noted that anxiety can exacerbate other symptoms, and treatment of anxiety by cognitive behavioral therapy can improve the social skills of patients.^20, 21^ Nrxn2α KO mice provide a tool to further explore the links between autism and anxiety. Nrxn1α KO mice exhibit milder anxiety-like behavior, making fewer transitions in a light/dark box^36^ while showing no abnormalities in the EPM.^37^ Other mouse models of autism have also shown anxiety phenotypes, including *Shank3* KO mice that spent less time in the open arms of the EPM.^38^

At the gene transcript level, we used quantitative RT-PCR to discover that various genes, normally associated with excitatory and inhibitory transmission, were downregulated in the Nrxn2α KO brain. However, only Munc18-1 showed detectable alterations at the protein level in western blotting assays. It is unclear why the decreased transcript levels of the other genes were not reflected in the abundance of their encoded proteins. Western blotting assays are, at best, only semi-quantitative, limited in their ability to detect small differences in protein levels. In accordance with the primary antibody suppliers' recommendations, total protein samples of 30 μg were loaded into each lane. Although loading less total protein or just the synaptosomal fraction might, conceivably, increase the sensitivity of western blotting, 40 μg of whole-brain homogenates were previously used to reveal the differences in synaptic proteins in *Nlgn1* KO mice deficient in neuroligin-1,^31^ a transmembrane protein that complexes with β-neurexin to form a functional excitatory synapse.^43^ Moreover, changes in gene expression level are frequently not reflected at the protein level;^44^ for example, a study using both RNA sequencing and quantitative mass spectrometry to calculate absolute mRNA and protein copy numbers in the same mouse fibroblasts found that mRNA levels explained only ~40% of protein level variation.^45^

A 21% decrease in the abundance of Munc18-1 in the brain has previously been found in *Nlgn1* KO mice that display impaired spatial memory and increased repetitive behavior.^31^ Interestingly, a genome-wide copy number variation analysis has implicated *NLGN1* as a candidate gene in autism susceptibility.^46^ A significant decrease in expression of Munc18-1 could conceptually be important for both excitatory and inhibitory transmission. Munc18-1 is found at the presynapse and holds a critical role in facilitating presynaptic vesicular release through its interactions with syntaxin-1.^34^ Furthermore, Munc18-1 can link to neurexins via its cytoplasmic tail through a complex that involves Mint1.^4^ In Munc18-1 KO mice, there is a complete loss of neurotransmitter secretion from synaptic vesicles^47^ by a reduction in docked vesicles at the active zone,^48^ whereas in heterozygotes the readily releasable pool is more easily depressed at glutamatergic and GABAergic synapses.^49^ Munc18-1 heterozygotes also show enhanced anxiety and impaired emotional learning.^50^ As altered Munc18-1 expression is likely just one of many synaptic modifications caused by the loss of Nrxn2α during development, further work is warranted to understand the molecular pathways that underpin the behaviors observed in Nrxn2α KO mice.

The robust deficit in social interaction and the heightened anxiety of Nrxn2α KO mice are consistent with a causal role for the loss of Nrxn2α in the genesis of autism-related behaviors, as suggested by the previous finding of deletions affecting exons of the *NRXN2* gene in two unrelated individuals with autism.^12, 13^ Nrxn2α KO mice may thus provide a useful experimental system for the exploration of disease mechanisms and novel treatments in autism.

## Figures and Tables

**Table 1:** Primer sequences for genes that were designed in-house

*Gene*	*Orientation*	*Sequence (5′*–*3′)*
*Cyc1*	Forward	ctctcctccttggaccacac
	Reverse	cggtacgccacataatcca
*Pgk1*	Forward	gaagcgggtcgtgatgag
	Reverse	attgtccaagcagaatttgatg
*Rpl13a*	Forward	ccctccaccctatgacaaga
	Reverse	gccccaggtaagcaaactt
*Grin2b*	Forward	tcatggtatcacgcagcaat
	Reverse	atcacccacacgtcagcac
*Stxbp1*	Forward	agaagaagggcgagtggaag
	Reverse	atcttgcagcaggaggacag
*Homer1*	Forward	ggccctctctcatgctagttc
	Reverse	ttgttgcctttgagggtagc
