# Supplementary material for: Deletion of α-neurexin II results in autism-related behaviors in mice
Source: Transl Psychiatry. 2014 Nov 25;4(11):e484–. doi: 10.1038/tp.2014.123 (PMC4259993; doi:10.1038/tp.2014.123)
Supplement: Supplementary Information [file tp2014123x1.doc]

# **Supporting Information**

**SI Materials and Methods**

All experiments were conducted in accordance with the United Kingdom Animals (Scientific Procedures) Act 1986, and performed under Project and Personal Licences held by the authors. All experiments were approved by the University of Leeds Ethical Review Process.

**Animals**

B6;129-*Nrxn3^tm1Sud^/Nrxn1^tm1Sud^/Nrxn2^tm1Sud^*/J mice (JAX # 006377) with a mixed (129X1/SvJ x 129S1/Sv)F1 and C57BL/6 background and black coat were purchased from the Jackson Laboratory (JAX) as heterozygous KO at *Nrxn1*, homozygous at *Nrxn2* and wild-type at *Nrxn3*. The line had been backcrossed at least twice (N2) to C57BL/6 (substrain unspecified) prior to deposition at JAX, where it was maintained by 8 generations of inbreeding prior to cryopreservation. Since the (129X1/SvJ x 129S1/Sv)F1 hybrid carries a deletion at *Disc1*, mice were genotyped using a PCR assay.^1^ Only mice wild-type at *Disc1* were outcrossed to the C57BL/6NCrl strain (Charles River) to segregate the *Nrxn1* and *Nrxn2* null mutations.

**Behavioral Experiments**

Mice were extensively handled for one week prior to commencing behavioral testing. All behavioral experiments commenced by transferring the mice to the experimental room 30 minutes prior to the start of testing. All apparatus was cleaned with 70% ethanol between subjects. Unless otherwise stated, all experiments were recorded using Any-Maze tracking software (Stoelting). The experiments were conducted in the order described below. For less stressful experiments, an interval of 2-3 days was provided between tests, while an interval of 1-2 weeks was given between more invasive tests (PPI, passive avoidance). For the social olfaction test and buried food test, a new cohort of animals was used that did not undergo any of the other tests.

**Open Field**

The open field arena had semi-transparent (frosted) Perspex walls, with an internal diameter of 40 x 40 cm. The floor of the arena was white plastic. A circular white outer wall (30 cm away from the arena wall) prevented the testing room from being visible during the trial. Lighting of the maze was measured using a lux meter at ~200 Lux at the center of the arena. A tripod supporting a webcam was positioned directly above the arena to record the trials.

Mice were placed into the arena facing the same wall for each mouse. They were left to explore undisturbed for 30 minutes. The tracking software divided the arena into the following zones, using the following dimensions: Outer Zone: 8 cm from the outer walls; Center Zone: 6.4 cm^2^ (16% of the total area); Intermediate Zone: the remaining area between the Outer Zone and the Center Zone (as defined by the European Mouse Phenotyping Resource of Standardised Screens [EMPReSS]). Time spent in, and entries to, each of the zones and distance travelled were measured. Rearing and grooming were measured during the trial by the experimenter.

**Elevated Plus Maze**

The elevated plus maze consisted of two closed arms with white opaque walls and two open arms. All arms measured 30 cm long and 5 cm wide. There was a central zone of 5 cm^2^ where all arms met. The mouse was then placed into the plus maze in the central zone, facing the opposing open arm from the experimenter. Mice were allowed to explore the maze for 5 minutes and were then removed and returned to the home cage. Mouse movement was tracked using Any-Maze. Time in and entries made to the open, closed and central zone were measured, in addition to the number of head dips over the side of the open arms. Entry into a zone was defined as when the hind legs crossed the boundary of the zone. The Any-Maze automated measurements were validated by offline manual scoring by an experimenter naïve to the experiment and with the data blinded (difference between Any-Maze and manual scoring: paired t-test (t_(33)_ <1, p > 0.05), data not shown).

**Forced Swim Test**

A 5L glass beaker (17 x 27 cm) was filled with 3L of water at 25 ± 1°C. The mouse was introduced into the water, facing away from the experimenter, and allowed to swim freely for 6 minutes. Time spent floating was measured from 2 minutes into the trial. Floating was defined as the lack of all movement except that required to keep the mouse afloat, but small limb movements that allowed the mouse to repeatedly swim into the side of the beaker were not counted as floating. After 6 minutes, the mouse was removed and was transferred to a cage under a heat lamp for around 20 minutes to warm gently.

**Tail Suspension Test**

To suspend the mouse, electrical tape was attached to the tail, near the tip, which was affixed to a structure that allowed the mouse to be suspended 20 cm from the ground. The trial lasted for 6 minutes, during which duration of immobility was measured. Immobility was defined as the lack of all movement except that required for respiration. At the completion of the trial, the mouse was removed and returned to the home cage.

**Social Interaction Test**

The protocol was adapted from Clapcote et al.^2^ Sociability was assessed using a three chambered maze (60 x 40 cm), which had two doors to allow the mouse to access the left and right chamber from the central chamber (each chamber being 40 x 20 cm, the door measuring 7 x 8 cm). Prior to testing, two stranger mice were thoroughly habituated to the apparatus by being placed into a cylinder, one cylinder being in the left chamber, and one in the right chamber. The cylinders measured 10 (w) x 10.5 (h) cm and consisted of vertical metal bars separated by 9 mm, allowing air exchange from the interior and the exterior of the cylinder and the possibility of contact between the test and stranger mice. During the test, the mouse (‘test mouse’) was placed into the center chamber and the doors were opened, allowing free access to all the chambers. A habituation period to the apparatus (without the cylinders) lasted for 15 minutes, at which point the test mouse was returned to the center chamber and the doors were closed. The cylinders were placed into the left and right chamber, and into one of them an unfamiliar male C57BL/6 mouse (age >10 weeks; ‘stranger 1’) was placed (left/right placement was counterbalanced across the group). The doors were then removed, allowing free access to both chambers for 10 minutes. Interactions of the test mouse with the stranger mouse and the empty cylinder were measured (counted as nose pokes within 2 cm of the cylinder or physical touches with the stranger mouse or the cylinder). At the end of the 10 minute period, the test mouse was returned to the center chamber and the doors were closed. The second stranger mouse (‘stranger 2’) was then placed into the empty cylinder, the doors were opened and the test mouse had a further 10 minute free exploration period, where interactions were with either stranger 1 or 2 were counted. Data were expressed as time spent exploring stranger 1 or the empty cylinder in the first stage, and time spent exploring either stranger 1 or 2 in stage two.

**Emergence Test**

The equipment consisted of a 40 x 40 cm open field arena (the same as for the open field test) containing a black enclosure (measuring 17cm L x 11cm W x 5.5cm H) placed against the middle of one arena wall (the positioning of the enclosure was alternated between trials). The enclosure had a 6 x 3.5 cm opening to allow the mouse free access between the enclosure and the arena. The trial commenced with the mouse being placed gently into the enclosure and the time was immediately started. Time spent out of the enclosure exploring the arena, latency to emerge from the enclosure, and the number of entries to the arena were all measured. Mice were allowed free exploration for 15 minutes.

**Novel Object Exploration**

Mice were first habituated to an open field arena (same as for the open field test). On days 1, 2 and 3, each mouse was placed into the empty arena for 15 minutes and allowed to explore freely. On day 4, mice were given a 10 minute habituation trial, after which they returned to the arena, which now contained two novel, but identical plastic objects (Mega Bloks, 6 x 6 x 4 cm (total) with four 2.5 x 2 cm cylinders on the top) placed 7 cm away from each opposing corner of one wall of the arena. The placement of the objects was varied during the experiment. Mice were allowed to explore the objects for 10 minutes, after which the mice were returned to their home cage. Exploration was defined as either direct contact (nose poke or touch) or exploration within close proximity (2 cm); accidental touches or behaviors such as sitting near the object were not counted. The equipment was cleaned with 70% ethanol after every stage of the experiment.

**Prepulse Inhibition**

Mice were tested for acoustic startle reactivity (ASR) and prepulse inhibition (PPI) using the SR-LAB startle response system (San Diego Instruments), which consisted of a sound attenuating box housing an animal enclosure platform, a fan and a speaker, all of which were lit from above by LEDs. Acoustic stimuli were delivered through a speaker at 70 dB, 80 dB, 85 dB, 90 dB and 120 dB, which was calibrated using a decibel counter (A weighting; Radio Shack). The animal enclosure platform that contained an accelerometer was calibrated before testing commenced using the San Diego Instruments Standardization Unit. Initial pilot data (not shown) suggested that a standardized value of 1000 mV from the platform was necessary to have sufficient measurable startle responses at low dB to measure ASR.

ASR and PPI were measured within the same trial, as outlined in the EMPReSS standard operating procedure (http://empress.har.mrc.ac.uk/viewempress/pdf/ESLIM_011_001.pdf). ASR was measured at ‘no sound’ (65 dB; BN; background noise), 70 dB, 80 dB, 85 dB, 90 dB and 120 dB using a single 40 ms pulse (‘no sound’ was a trial with only the background noise audible). PPI was measured by the delivery of a tone at either 70 dB, 80 dB, 85 dB, 90 dB (prepulse) for 10 ms followed by a 100 ms gap at background noise and then a 120 dB ‘startle’ tone for 40 ms. During the trials, background noise at 65 dB presented throughout. Intra-trial intervals were averaged at 25 seconds.

A trial consisted of a 5 minute habituation period, during which the 65 dB noise was presented. After this, five 120 dB startle pulses were delivered, although these were subsequently excluded from the analysis. PPI and ASR were then tested in 10 blocks, of which each block contained 10 trials (ASR: ‘no sound’, 70 dB, 80 dB, 85 dB, 90 dB and 120 dB; PPI: 70 dB, 80 dB, 85 dB, 90 dB). The delivery of the tones was pseudorandom and the pattern was never repeated across blocks. Startle responses were detected by an accelerometer on the platform, digitized and saved to computer in waveform format, with a post-stimulus time window of 300 ms and resolution of 1 kHz.

ASR was analyzed by measuring the V_max_ of the mV waveform from 0 to 100 ms. ASR was then averaged for all 10 trials at each dB intensity. PPI trials were analyzed using the same method, except that the waveform was measured in response to the second, ‘startle’ pulse from 120 to 220 ms. mV values were again averaged for each dB intensity. PPI was calculated as (mean 120 startle alone – mean PPI db startle) / mean 120 startle alone x 100, where mean PPI dB startle represents a separate calculation at each of the 70 dB, 80 dB, 85 dB, 90 dB PPI trials.

**Passive Avoidance**

Passive avoidance was assessed using a Med Associate Shuttle Box under the control of MedPC software. The shuttle box consisted of two chambers divided by a remotely operated guillotine door, housed within a sound attenuation box with a fan providing background noise. One half of the chamber was darkened using a black cloth cover (defined as the ‘dark chamber’). The other side of the chamber was lit by a white light. The conditioning trial commenced with the door closed. The mouse was placed into the light side of the chamber, and after a 10 second delay, the door to the dark chamber opened. Latency for the mouse to pass to the dark chamber was measured. When the mouse fully entered the dark chamber, the door closed and after 10 seconds, one 3-second 0.45 mA footshock was delivered. The mouse was removed 30 seconds thereafter. 24 hours later, the latency to cross to the dark chamber was measured. The process was the same as for the conditioning, except that no footshock was delivered if the mouse crossed to the dark chamber. The trial was stopped either if the mouse had not crossed within 300 seconds, at which point the door closed and the mouse was removed (with a time of 300 seconds attributed to that mouse), or when the mouse had traversed to the dark chamber.

**Social Olfaction Test**

The apparatus and running of this test were the same as for the social interaction test, except there was no ‘stage 2’ for social discrimination. Soiled bedding was obtained from a cage containing 4 adult male mice that had inhabited the cage for 1.5 weeks after fresh bedding was introduced. All mice were exposed to the same soiled bedding. The bedding was put into an open petri dish (3.5 x 1 cm) and placed into the middle of the cylinders, which were the same as used for the social interaction test.

**Buried Food Test**

Mice were trained to consume a chocolate flavored cereal reward (Wheetos, Weetabix) by placing the reward into the home cage for 2 days prior to testing. On the evening before testing, the normal food was removed, and samples of the reward were scattered within the cage. For the test, the mouse was transferred to a clean test cage containing 3 cm of fresh bedding and allowed to habituate for 5 mins. Following habituation, the mouse was put into a clean holding cage without bedding. A single food reward was buried to a depth of 1 cm in the test cage. The mouse was then returned to the test cage and allowed to find the buried reward. The experiment was terminated after 15 mins if the mouse did not locate the food, and a score of 900 s was given.

**Results**

**Figure S1**

**
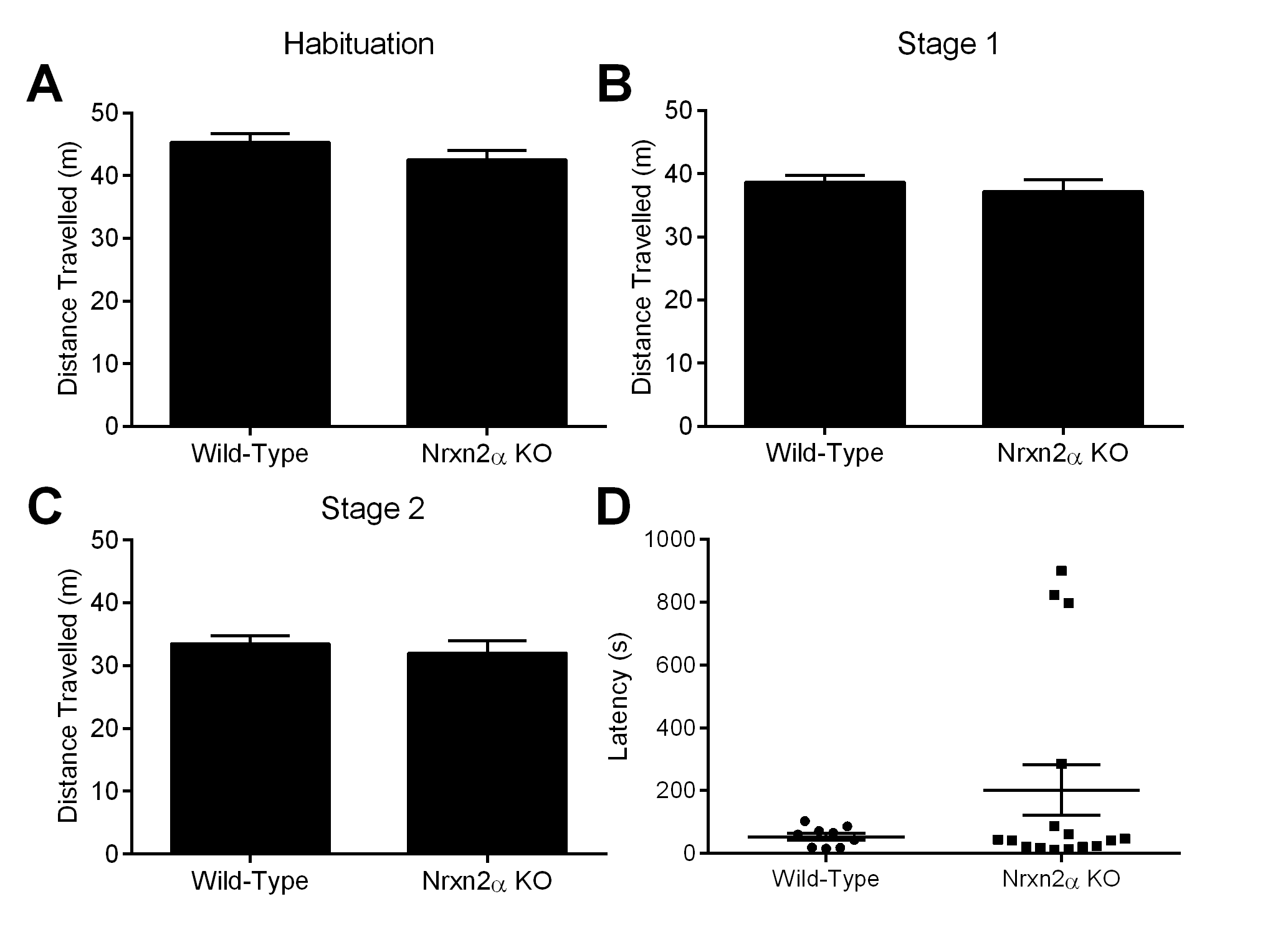
**

**Figure S2**

**
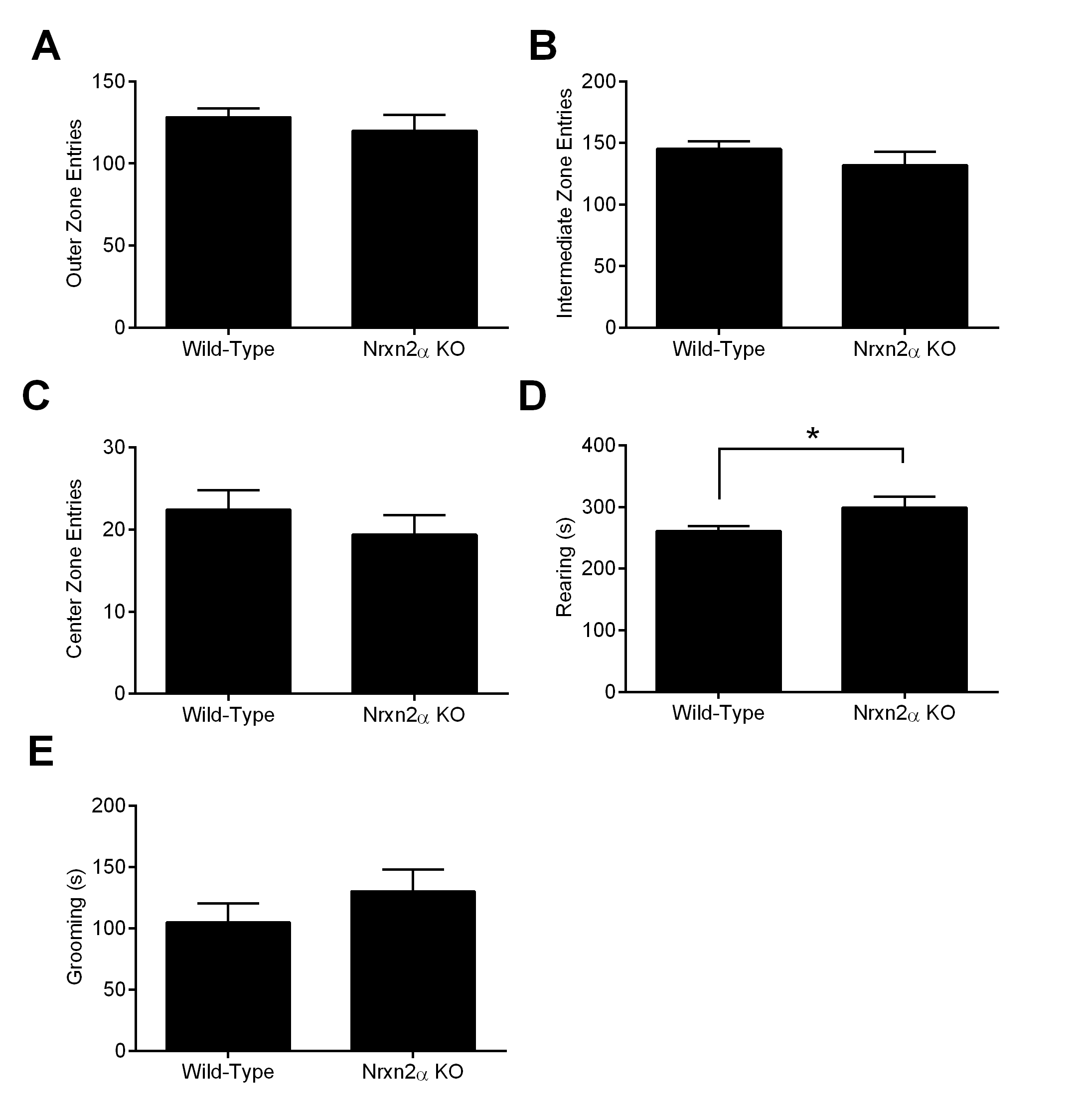
**

**Figure S3**

**
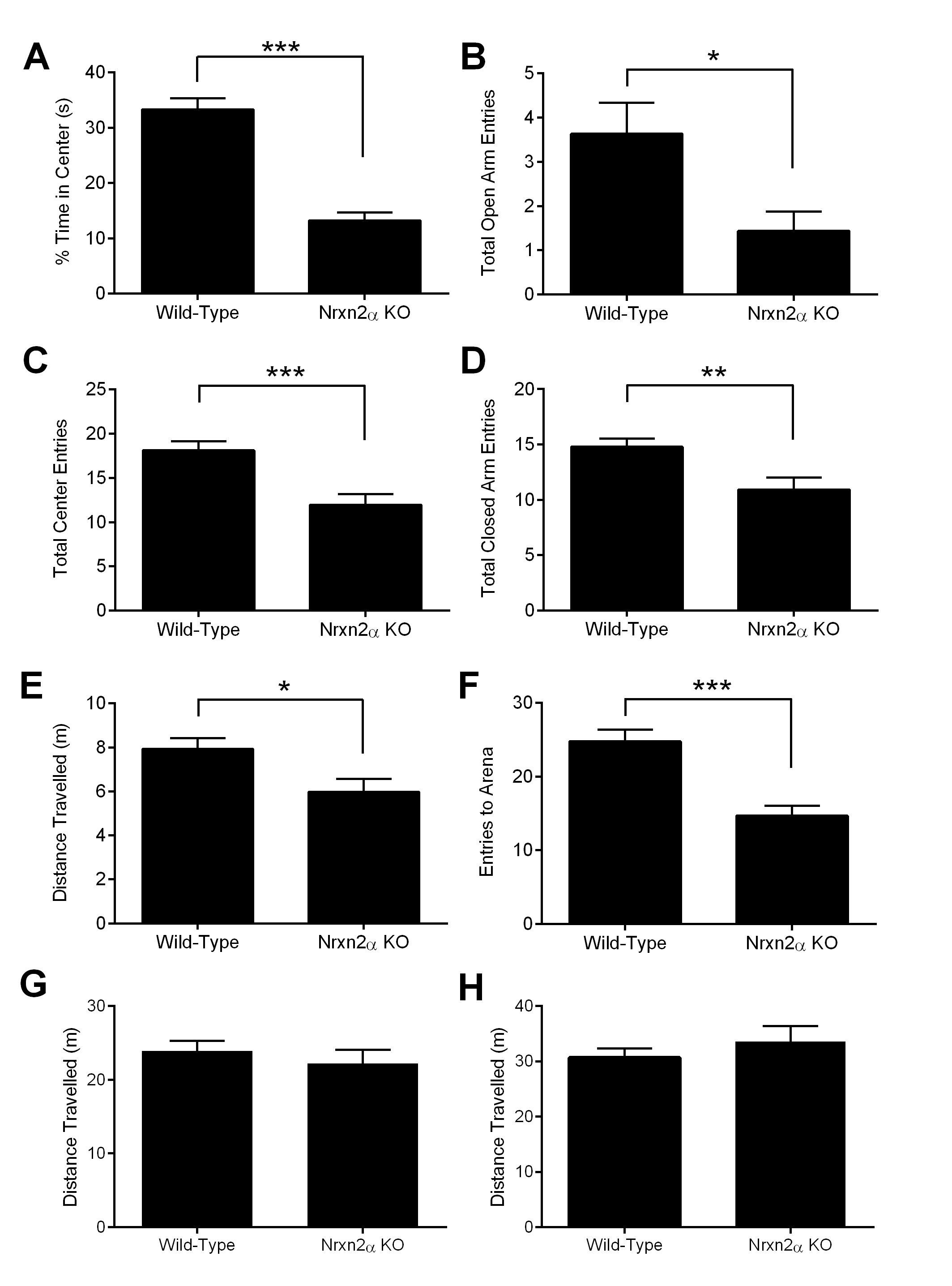
**

**Figure S4**

**
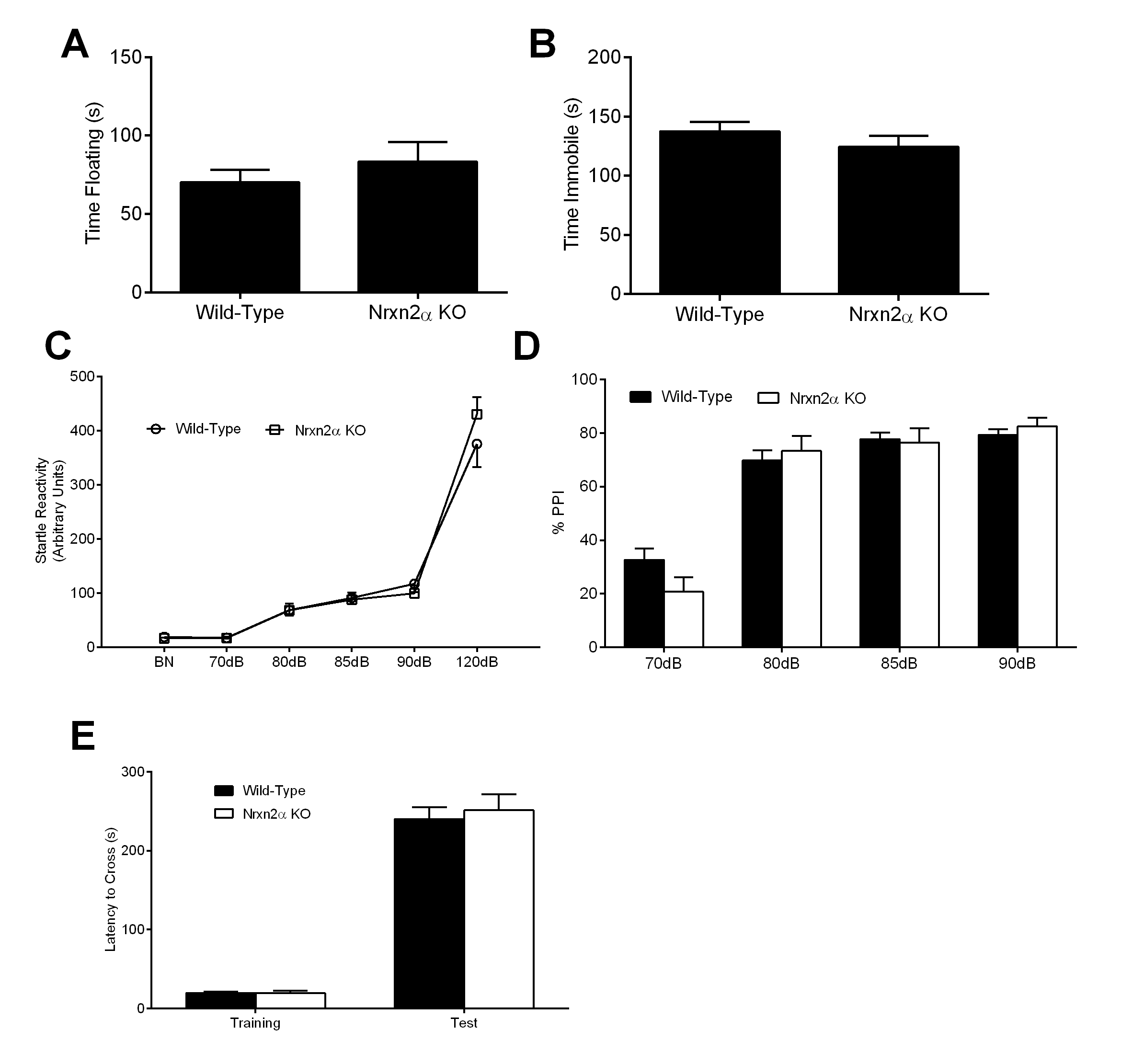
**

**Figure S5**

**
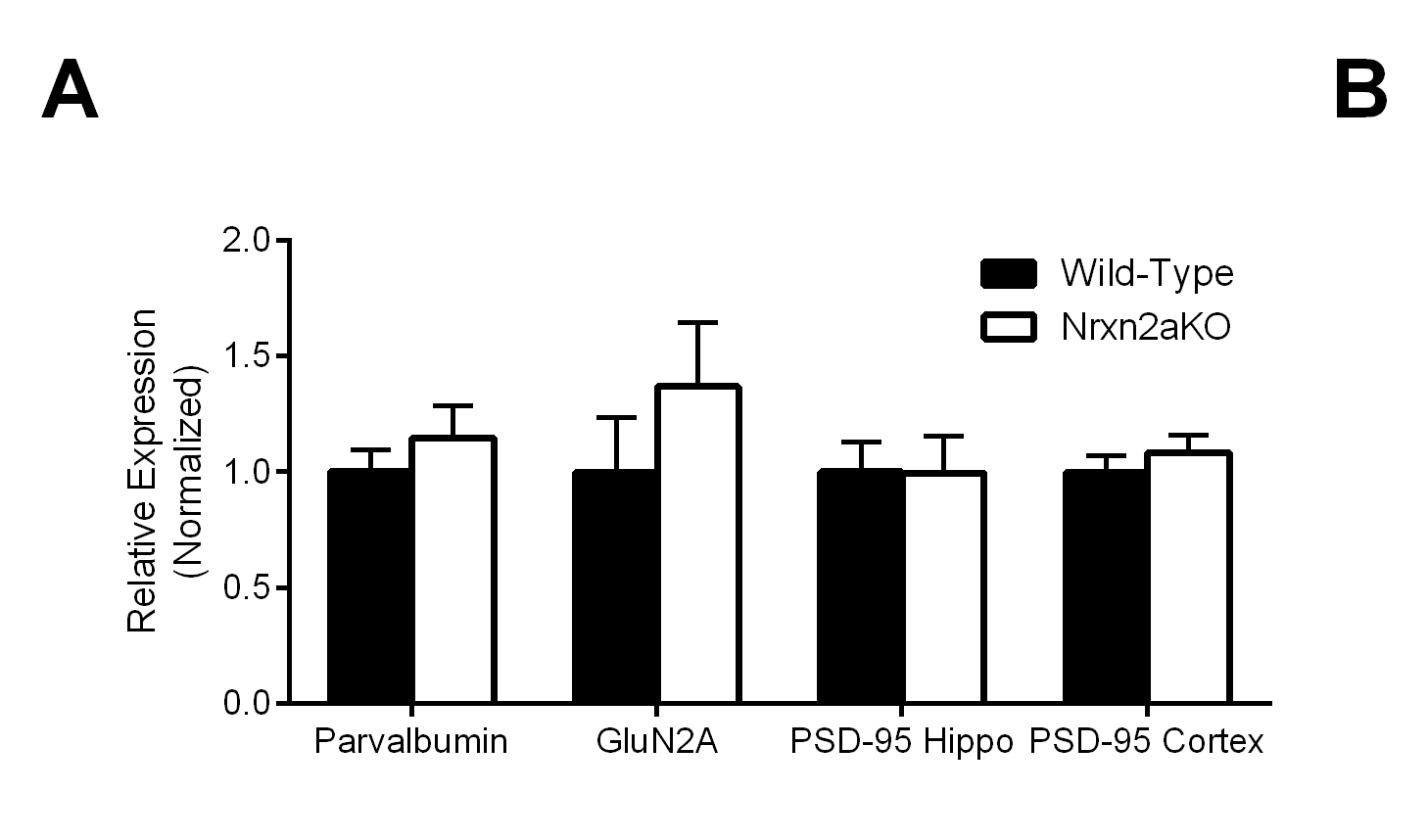
**


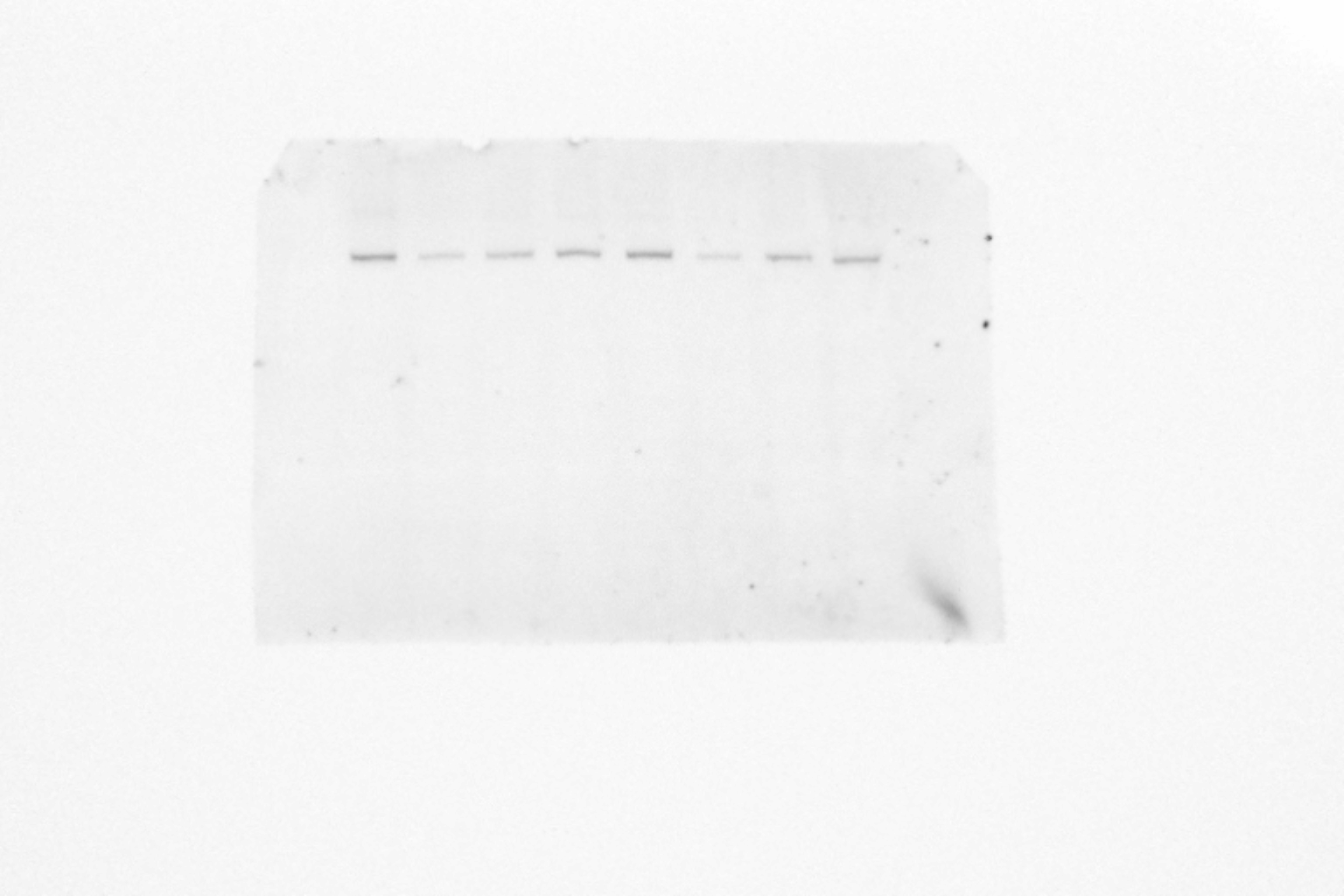


GluN2A Hippocampus


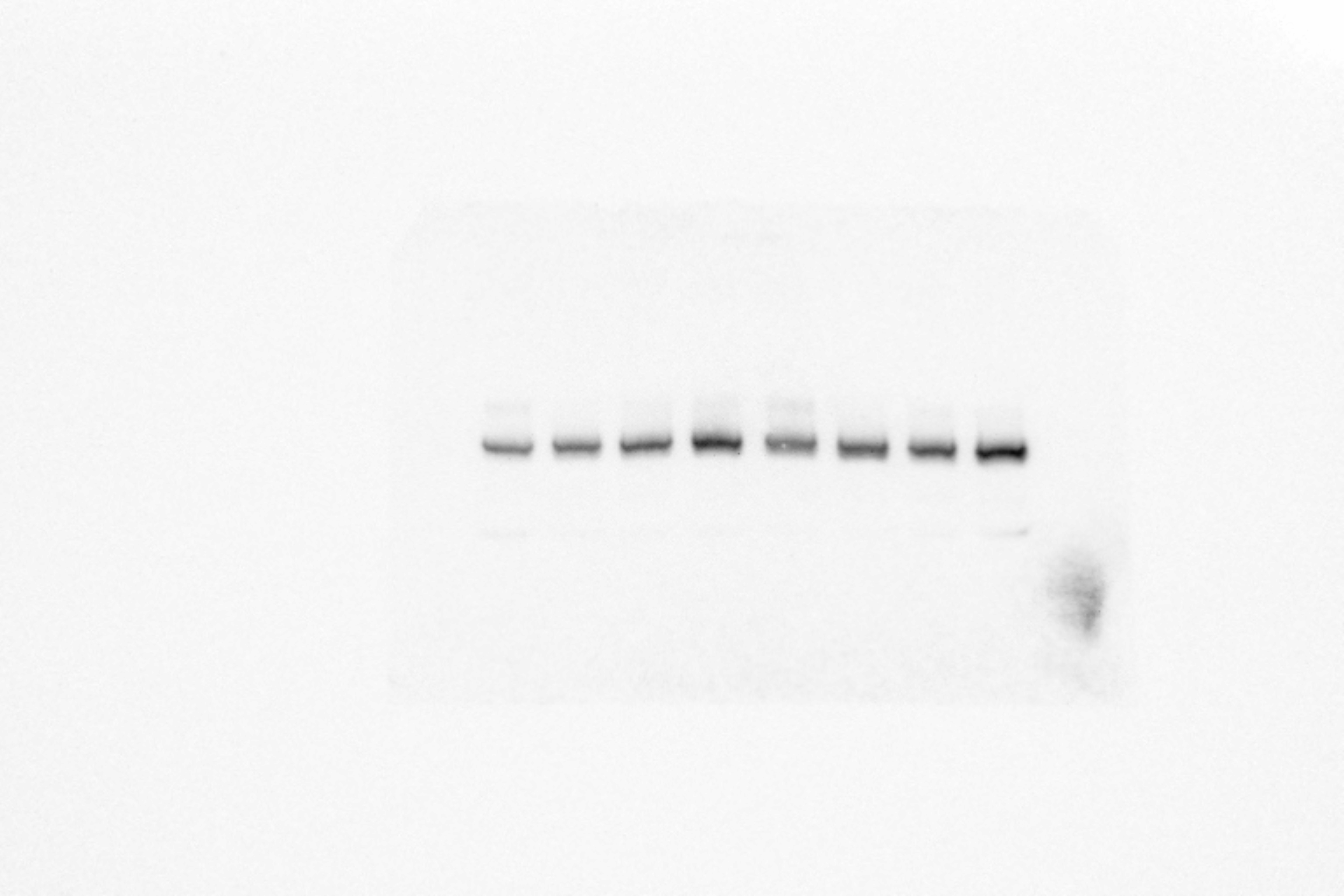


PSD-95 Hippocampus


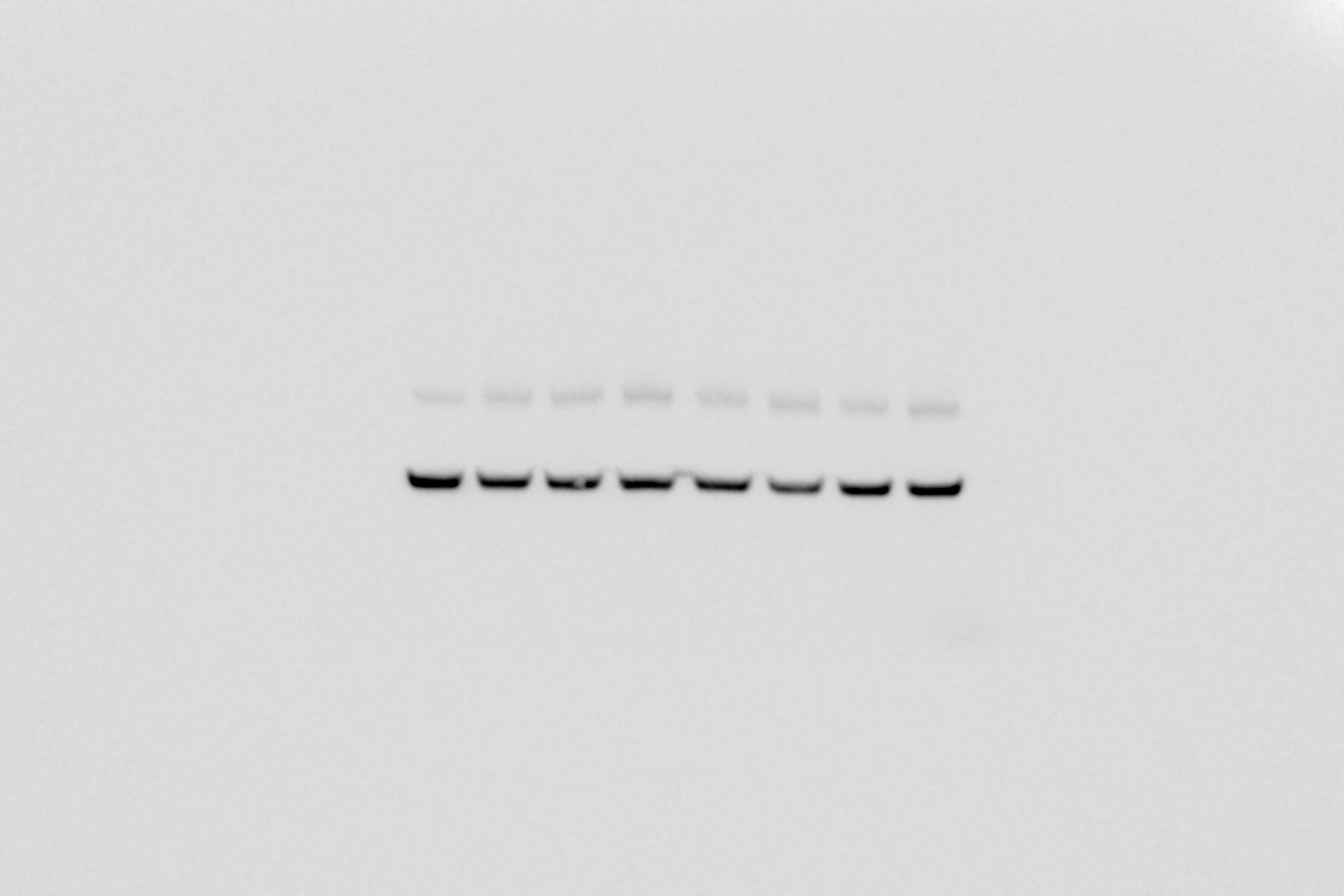


β-actin Hippocampus

WT

KO


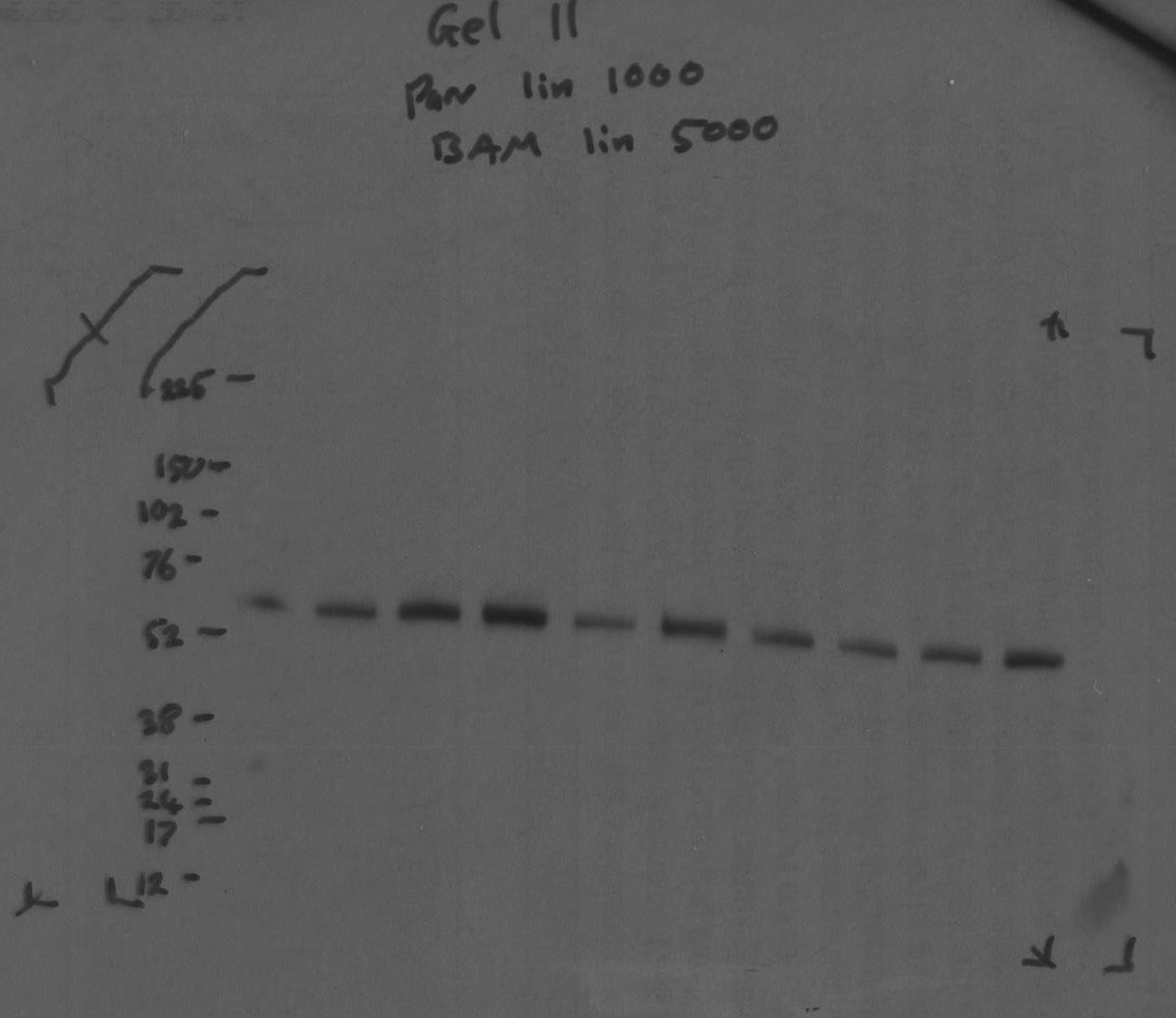

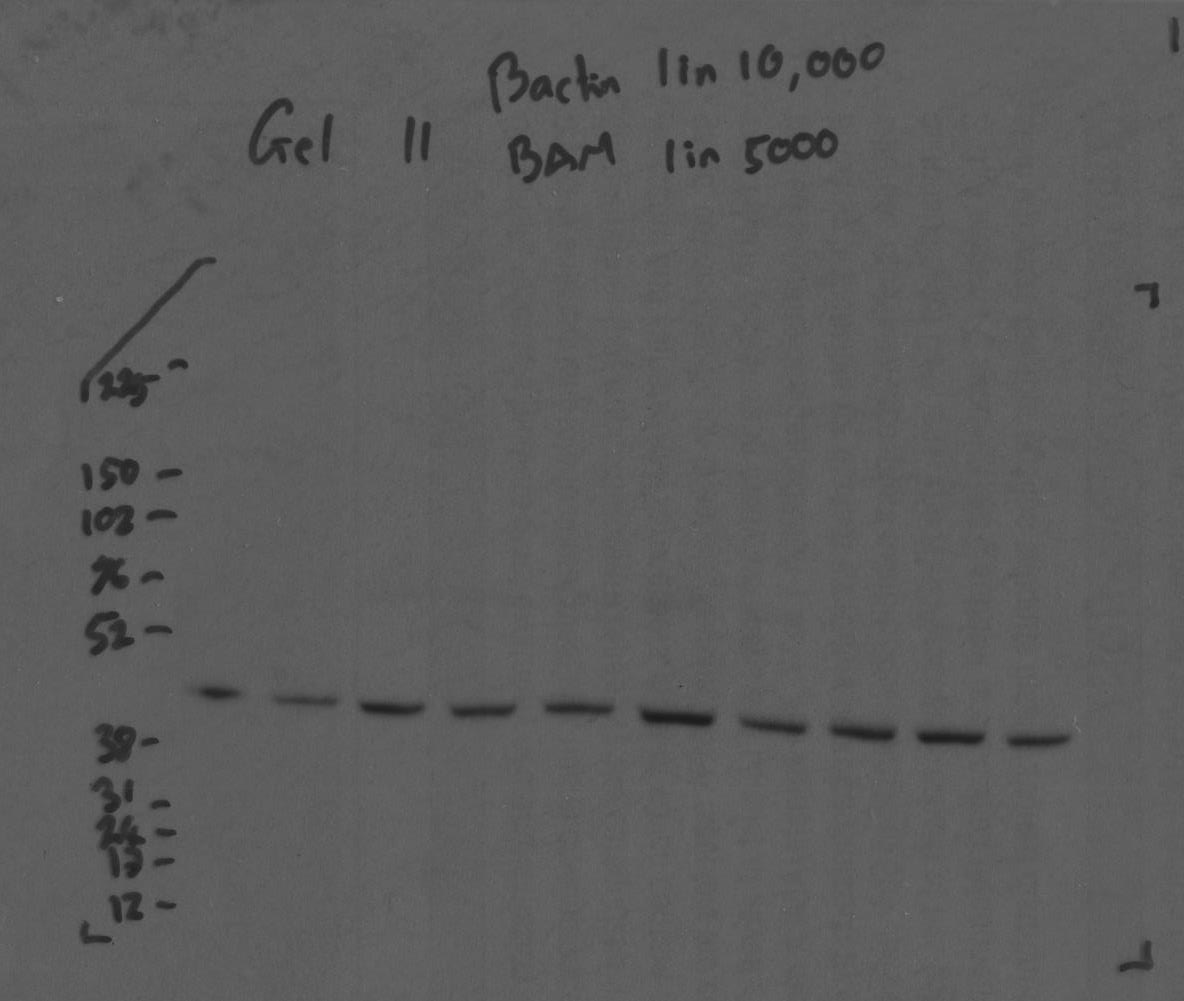


Pvalb Hippocampus

β-actin Hippocampus


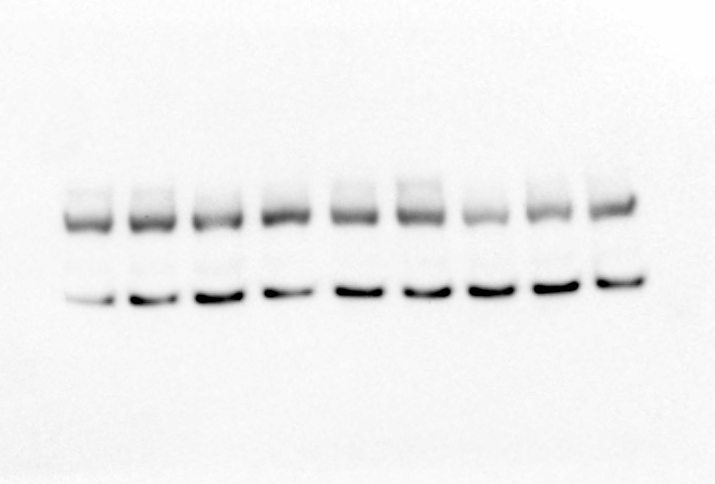

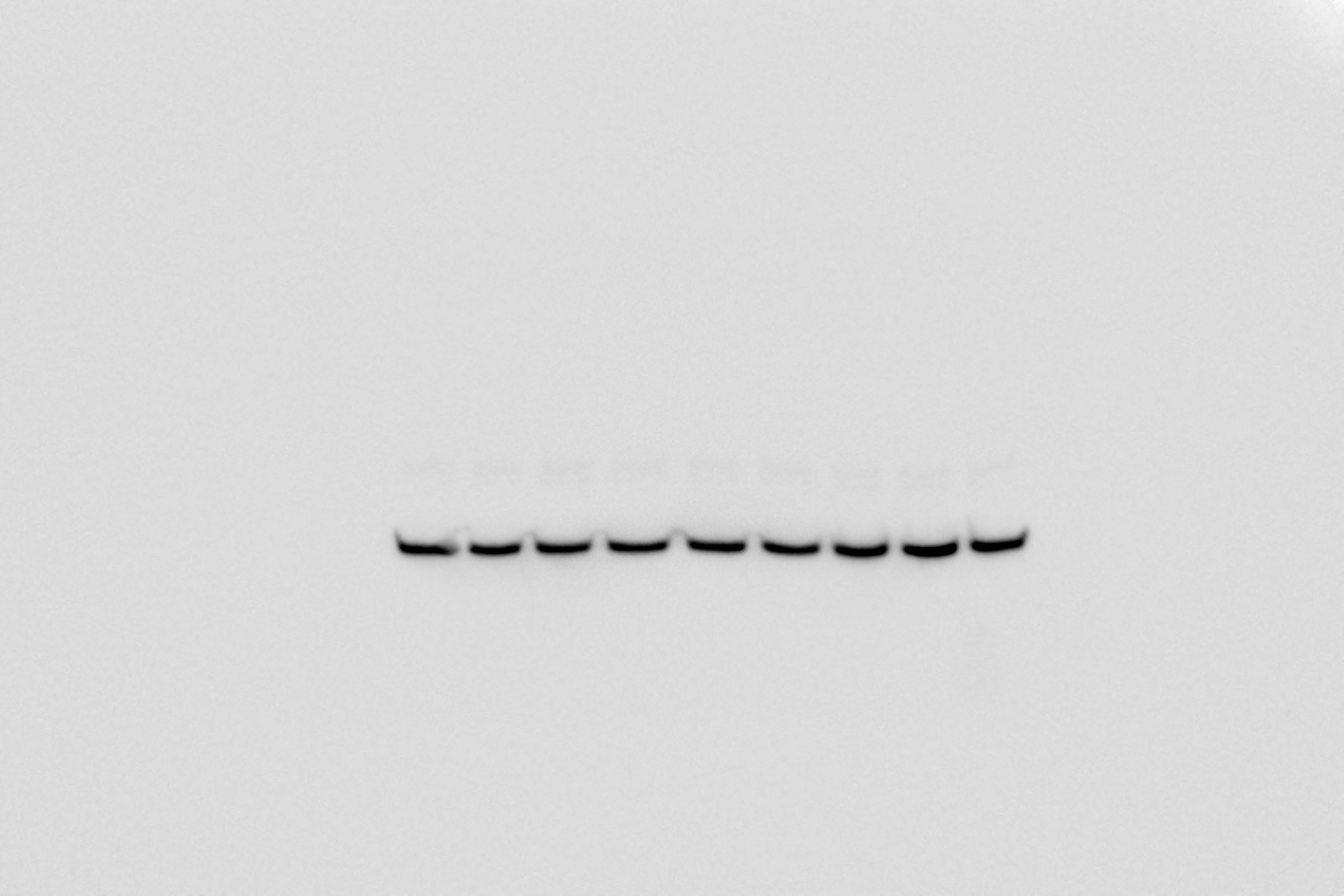


PSD-95 Cortex

β-actin Cortex

WT

KO

**Supp. Figure Legends**

**Figure S1**

Sociability and social memory in WT and Nrnx2α KO mice. During a 15-minute habituation period (A), mice of both genotypes travelled a similar distance (unpaired t-test, t_(45)_ = 1.27, p > 0.05), similar to the results from the open field arena (Fig. 2A). During stage 1 (B) and stage 2 (C) of the experiment, in which the test mouse had to discriminate between a novel mouse and an empty cage and then between the previously explored mouse and a second novel mouse, the total distance travelled was similar between the genotypes (t_(45)_ = 0.78, p > 0.05 and t_(45)_ = 0.65, p > 0.05, respectively). To determine whether a lack of olfaction could explain the deficits in social responses in Nrnx2α KO mice, their ability to locate a buried food reward was tested (D). No significant differences were observed between the genotypes (unpaired t-test, t_(23)_ = 1.36, p > 0.05; WT *n* = 9, KO *n* = 16).

**Figure S2**

Activity within the open field. Tracking software measured the number of entries made into three zones (outer (A), intermediate (B) and center (C)) by WT and Nrnx2α KO mice during the 30 minute trial. While the KO mice spent a significant proportion of their time in the outer but not the intermediate zones (see Fig. 1), there were no significant differences between the genotypes for the total entries made into any of the three zones (outer zone; t_(47)_ = 0.80, p > 0.05, intermediate zone; t_(47)_ = 1.11, p > 0.05, center zone; t_(47)_ = 0.81, p > 0.05). While Nrnx2α KO mice spent a significantly greater time rearing (D; t_(47)_ = 2.31, p = 0.03), the time spent self-grooming, a behavior thought to be reflective of the repetitive behaviors associated with autism, was similar to WTs (E; t_(47)_ = 1.01, p > 0.05).

**Figure S3**

Measures of anxiety in Nrnx2α KO mice. Within the elevated plus maze, Nrnx2α KO mice spent significantly less time in the center platform (A; t_(47)_ = 6.52, p < 0.0001), and made significantly fewer entries to the open arms (B; t_(47)_ = 2.10, p = 0.04), the center (C; t_(47)_ = 3.57, p = 0.0008) and the closed arms (D; t_(47)_ = 2.98, p = 0.0045) compared to WT controls. The KO mice also travelled less within the maze (E; t_(47)_ = 2.38, p = 0.021), which is likely to be an anxiety-driven behavior. During the emergence test, in corroboration with the reduced total time spent out of the enclosure (Fig. 3E), KO mice made significantly fewer entries to the open arena from the enclosed shelter (F; t_(47)_ = 3.96, p < 0.0001). Locomotion was not different between the genotypes in the novel object test, neither during the habituation period prior to object exposure (F; t_(47)_ = 0.94, p > 0.05) nor the object exposure period itself (G; t_(47)_ = 0.72, p > 0.05).

**Figure S4**

Nrnx2α KO mice are unaffected in measures of despair, sensory gating and cognition. In two tests of behavioral despair, the Porsolt forced swim test (A) and tail suspension (B), Nrnx2α KO mice showed no significant differences in the time spent floating or immobile, respectively (t_(47)_ = 0.93 and t_(47)_ = 1.01, both p > 0.05). The acoustic startle reactivity (C) to a variety of tones ranging from background noise (BN; 65 dB) to 120 dB showed no difference between the genotypes (RM ANOVA, genotype (F_(1, 47)_ <1, p > 0.05), tone intensity (F_(5, 235)_ = 106.4, p < 0.0001), genotype by tone intensity interaction (F_(5, 235)_ <1, p > 0.05)). PPI (D) was similarly unaffected by Nrnx2α KO (RM ANOVA, genotype (F_(1, 47)_ <1, p > 0.05), pre-pulse intensity (F_(3, 141)_ = 194.3, p < 0.0001), although there was a significant interaction between genotype and pre-pulse intensity (F_(3, 141)_ = 3.99, p = 0.009)). Despite the significant interaction, tests of simple main effects failed to reveal significant differences at any of the pre-pulse intensities (70 dB, F_(1, 47)_ = 2.90, p = 0.095; 80, 85 and 90 dB, all F_(1, 47)_ <1, p > 0.05). The passive avoidance test, which requires a mouse to associate a footshock with a context to inhibit their natural instinct to cross from a lit chamber to a dark chamber, also failed to highlight any genotypic differences (RM ANOVA, genotype (F_(1, 47)_ <1, p > 0.05), latency to cross following training (F_(1, 47)_ = 323.6, p < 0.0001), interaction between the factors (F_(1, 47)_ <1, p > 0.05)), suggesting that cognition was unaffected in Nrnx2α KO mice.

**Figure S5**

Protein expression of genes identified as significantly altered by RT-PCR. Homogenates from wild-type and Nrnx2α KO mice were probed with antibodies for PSD-95 (hippocampus and cortex), GluN2A (hippocampus) and Pvalb (hippocampus). No significant differences were observed for any protein or brain area (Pvalb t_(28)_ <1, p > 0.05; GluN2A t_(22)_ = 1.01, p > 0.05; hippocampal PSD-95 t_(22)_ <1, p > 0.05; cortex PSD-95 t_(25)_ <1, p > 0.05).

**References**

1. Clapcote SJ, Roder JC. Deletion polymorphism of Disc1 is common to all 129 mouse substrains: implications for gene-targeting studies of brain function. *Genetics*. 2006; 173 (4): 2407-10.
2. Clapcote SJ, Lipina TV, Millar JK, Mackie S, Christie S, Ogawa F *et al*. Behavioral phenotypes of Disc1 missense mutations in mice. *Neuron*. 2007; 3; 54 (3): 387-402.
